# Adult Laryngeal Coin Impaction Presenting With Isolated Dysphonia: A Report of a Rare Case

**DOI:** 10.7759/cureus.102881

**Published:** 2026-02-03

**Authors:** Ridhima Malik, Ditixa Patel, Nikhil Arora, Deepika Saini, Yogender Yadav

**Affiliations:** 1 Department of Otolaryngology, Head and Neck Surgery, Maulana Azad Medical College, Delhi, IND

**Keywords:** accidental, coin ingestion, dysphonia, dyspnea, foreign body larynx

## Abstract

Impaction of a foreign body in the larynx is commonly seen in children but remains a rare occurrence in adults. Laryngeal impaction, particularly of a coin, is exceedingly uncommon and carries a potential risk of airway compromise. Prompt history taking, clinical examination, and radiographic evaluation are essential for diagnosis. We report a rare case of an adult male with a laryngeal coin impacted sagittally between the true vocal cords, presenting solely with isolated dysphonia and without dyspnea or stridor. This case is atypical due to adult presentation, minimal symptoms despite glottic involvement, and sagittal orientation of the foreign body, highlighting the importance of maintaining a high index of suspicion for laryngeal foreign bodies, even when airway symptoms are minimal.

## Introduction

Accidental aspiration of foreign bodies is frequently observed in children; however, it remains a rare event in the adult population. Among these rare cases, laryngeal impaction, particularly by a coin, is exceptionally uncommon, with very few reports documented in the literature [1]. Symptoms can range from dysphagia and odynophagia to hoarseness and breathing difficulty, persistent cough, and stridor, depending upon the site and orientation of the foreign body impaction. Adult laryngeal foreign bodies may present with subtle or atypical symptoms depending on the size, orientation, and anatomical level of impaction. Glottic foreign bodies positioned in the sagittal plane may permit partial airway patency, resulting in isolated symptoms such as dysphonia and delayed presentation, thereby increasing the risk of missed or delayed diagnosis. Delayed removal can lead to complications such as airway oedema and life-threatening conditions [2]. 

We present a rare case of a 51-year-old male who ingested multiple coins, one of which became impacted between the vocal cords. Notably, presenting solely with hoarseness of voice and without any respiratory compromise. Because of the potential for sudden airway compromise, laryngeal foreign bodies constitute an otolaryngologic emergency even in the absence of overt respiratory distress. Diagnosis was established through clinico-radiological evaluation. The foreign body was successfully extracted via endoscopic intervention. This case highlights the importance of maintaining a high index of suspicion for laryngeal foreign bodies, even in the absence of classic respiratory symptoms.

## Case presentation

**Figure 1:**
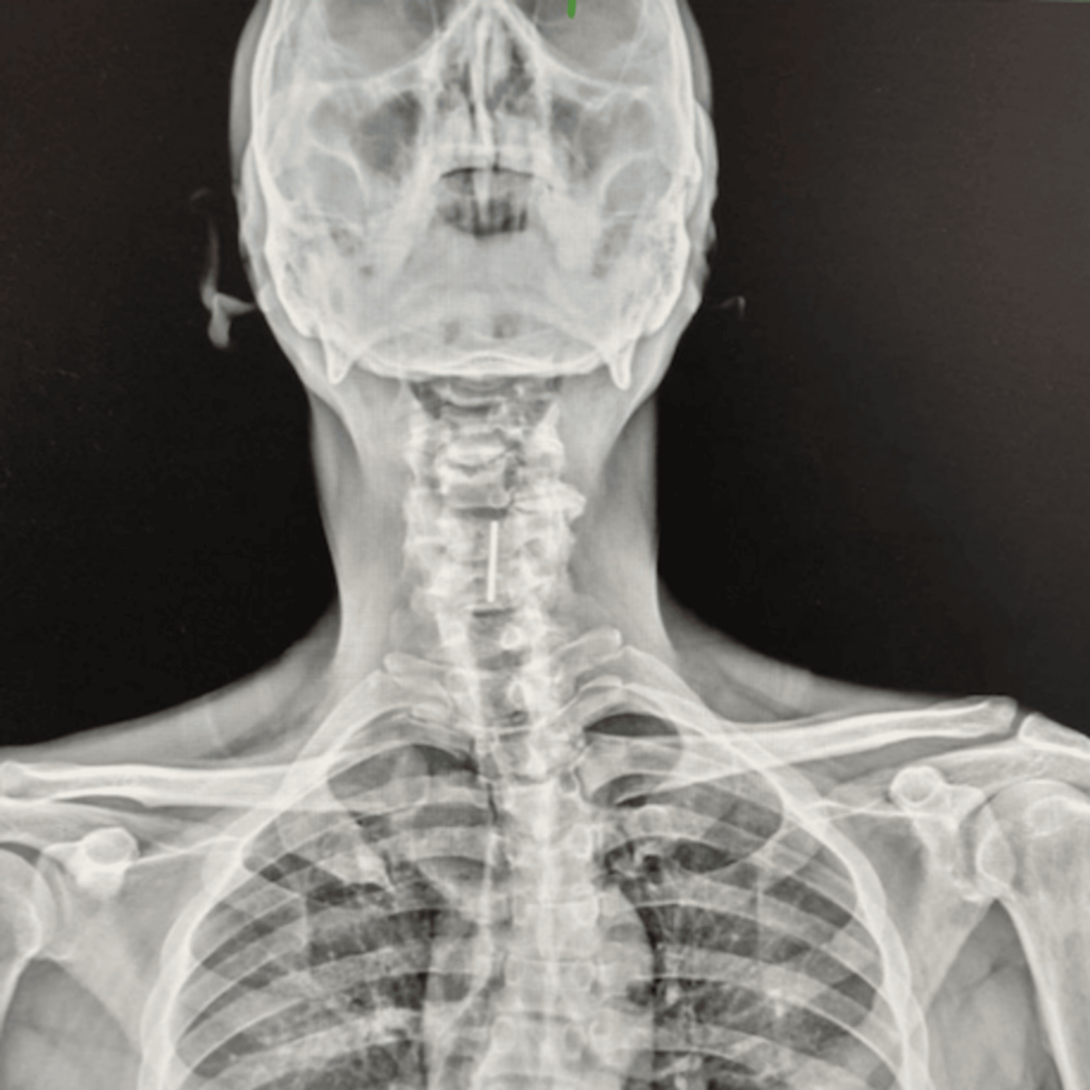
X-ray neck (anteroposterior view) The image is showing a radio-opaque foreign body (white thick line).

**Figure 2:**
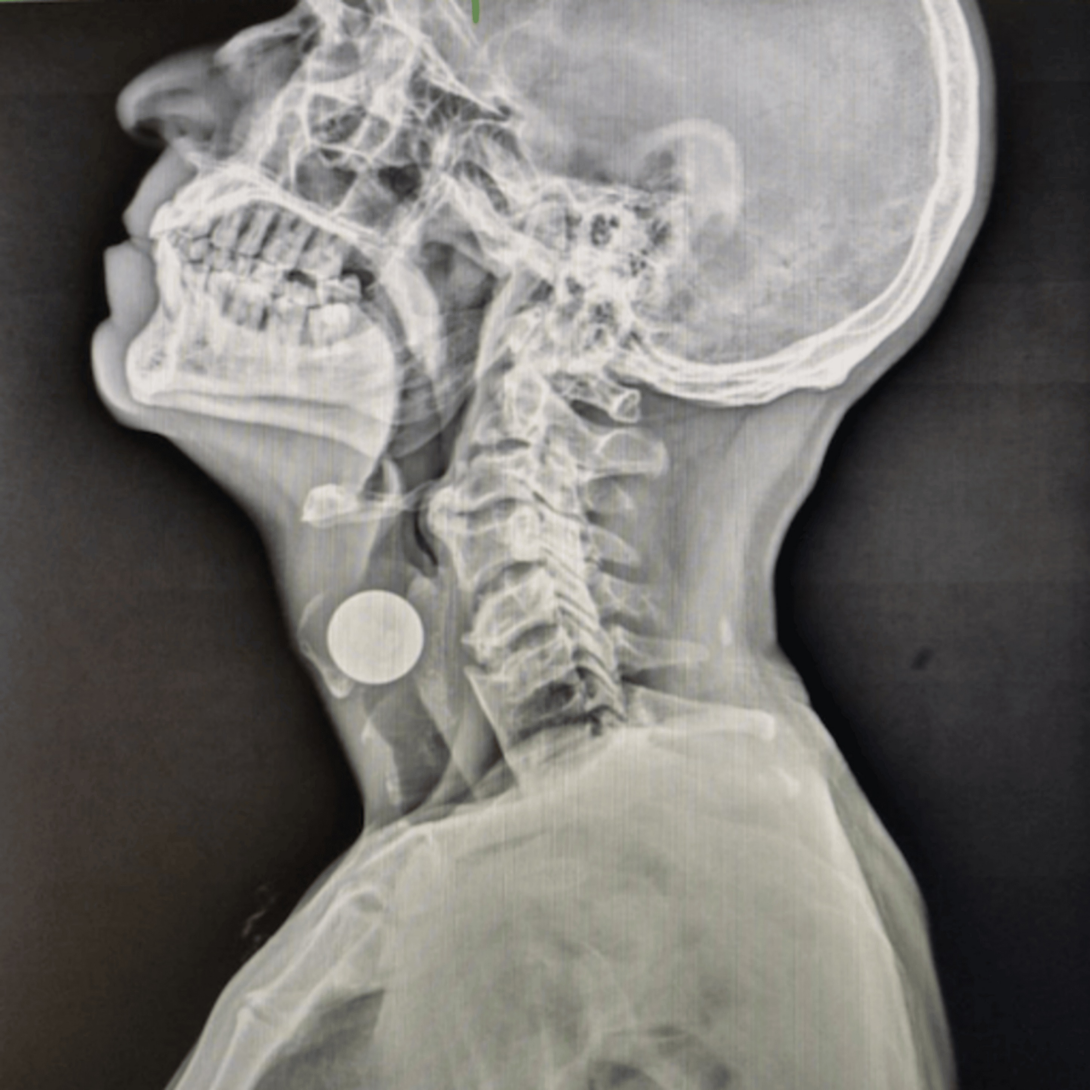
X-ray neck (lateral view) The image is showing a round radio-opaque foreign body in the airway at the level of the glottis (white opaque circle).

A 51-year-old male presented to the ENT outpatient department with complaints of persistent hoarseness of voice for four days. He denied any difficulty swallowing, pain, stridor, dyspnoea, cough, or haemoptysis. On further inquiry, the patient revealed that he had ingested multiple coins four days ago during an episode of binge drinking, but did not seek immediate medical attention. On further assessment, there was no past history of psychiatric illness, intellectual impairment, neurological disease, or deliberate self-harm, and the ingestion was found to be accidental and occurred in the context of acute alcohol intoxication. On rigid laryngoscopy using a 70º Hopkins telescope, the coin was lodged vertically between the two vocal cords. A neck X-ray (anteroposterior and lateral views) demonstrated a radiopaque circular foreign body at the level of the glottis (Figures 1, 2).

**Figure 3:**
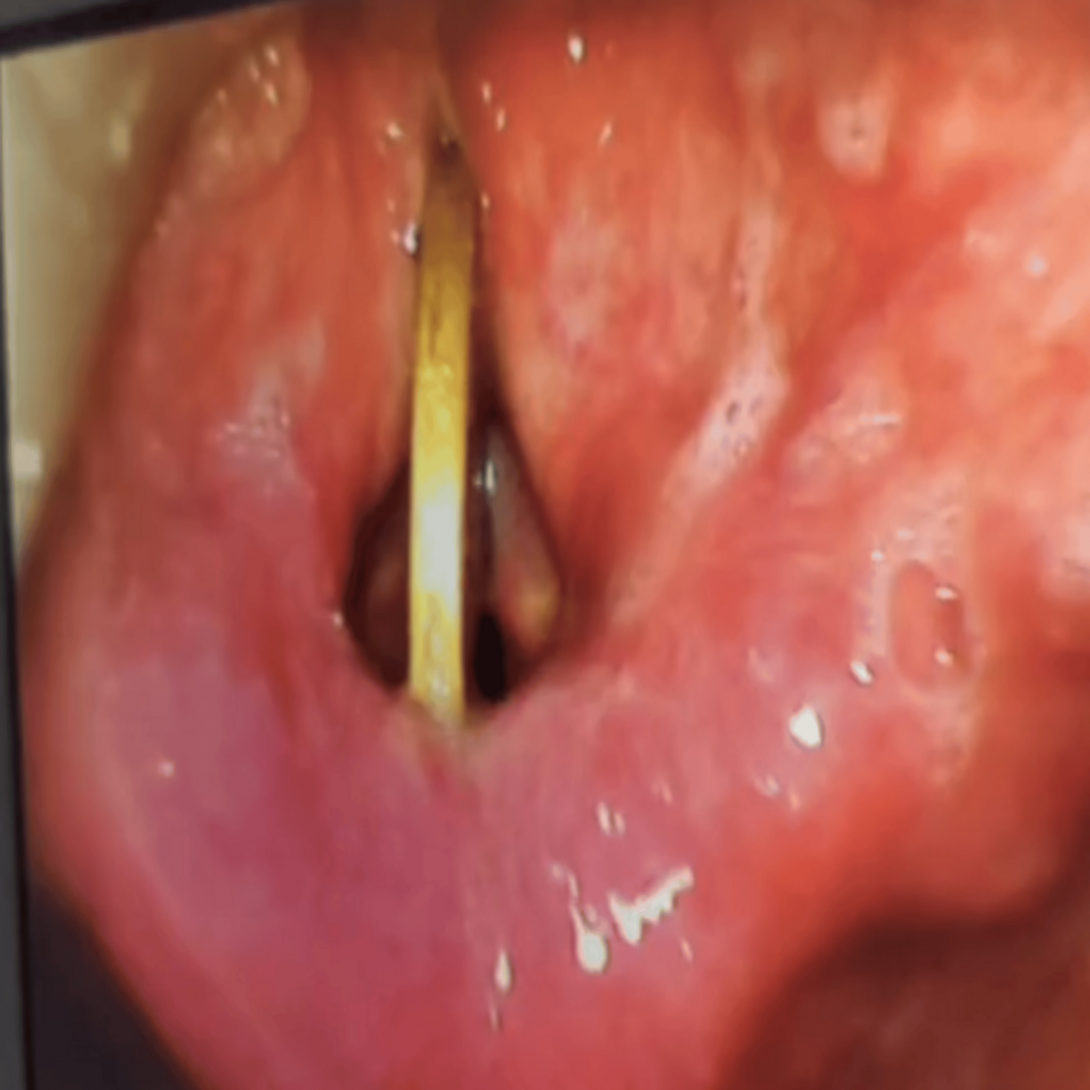
Intraoperative endoscopic view The image is showing a coin impacted vertically between the true vocal cords.

Chest and abdominal radiographs were obtained simultaneously to evaluate for additional ingested coins and revealed multiple metallic foreign bodies in the gastrointestinal tract, which subsequently passed through the stool without any complications. In view of the risk of sudden airway obstruction, the patient was taken up for urgent direct laryngoscopy. Removal of the coin was performed under intravenous sedation with maintenance of spontaneous ventilation; neuromuscular blockade was deliberately avoided to prevent loss of airway tone and minimise the risk of distal migration of the foreign body (Figure 3).

**Figure 4:**
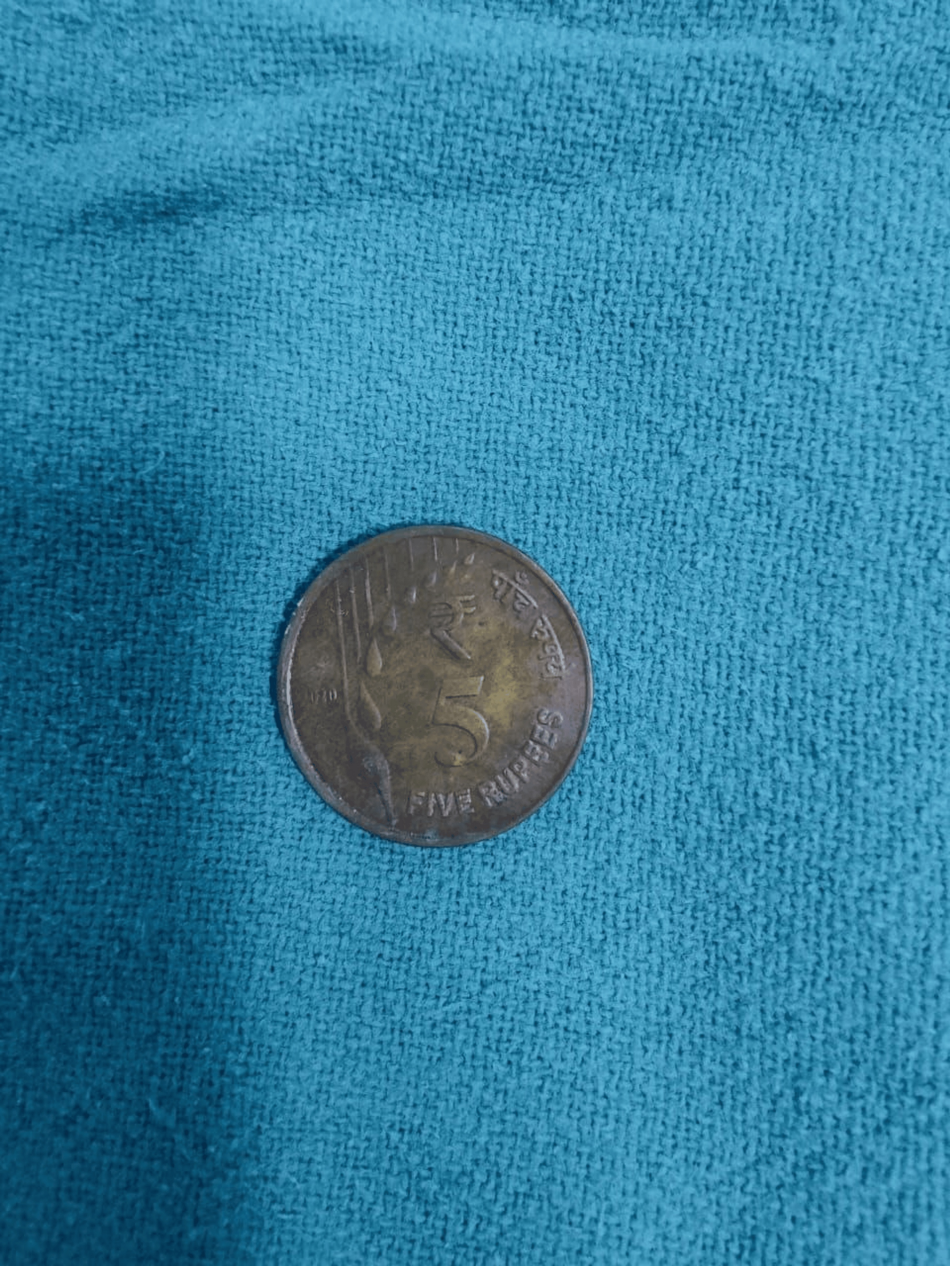
Removed laryngeal coin following endoscopic extraction

The coin was carefully extracted under direct visualisation without complications (Figure 4). Immediately after removal, post-procedural direct laryngoscopy confirmed intact mucosa and normal vocal cord mobility. The patient’s voice improved immediately after the procedure. He was discharged the next day with advice for follow-up after one week, during which he remained asymptomatic.

## Discussion

Laryngeal foreign body (FB) impaction is uncommon, representing approximately 2-9% of cases in adults. Most of these instances involve inorganic objects [3]. Laryngeal foreign body impaction is more frequently observed in children, who usually present with sudden respiratory distress, with the object most often located in the trachea or bronchi [4]. Foreign body aspiration is particularly common at the extremes of age. The highest incidence of foreign body aspiration is in children younger than three years, and a second peak occurrence is seen in adults older than 50 years [5-7]. Foreign body aspiration is a condition that mainly occurs in the extremes of age. While in children, the susceptibility is mainly due to the immaturity of the coordination of swallowing and the ineffective cough reflex, in adults, the predisposing conditions are more likely to be linked to foreign body aspiration, such as advanced age, neurological damage, altered sensorium from alcohol intoxication, psychiatric disease, or trauma [7].

In adults, the aspirated foreign bodies are most likely to enter the bronchi, followed by the trachea, and the larynx is a distinctly rare site of impaction [8]. The symptoms associated with foreign body impaction differ according to the anatomical site involved. Supraglottic foreign bodies often lead to symptoms such as dysphagia and odynophagia. When located in the glottic region, patients may exhibit hoarseness and cough. Subglottic impaction generally presents with breathing difficulty, persistent cough, and stridor. Hada et al. [9] explained the absence of respiratory distress in glottic foreign bodies by the sagittal position of the coin, causing only a partial obstruction, which can lead to a delay in consultation, as happened in our patient as well. Reported delays in presentation range from one to three days [1,9]. Delayed removal can lead to complications ranging from airway oedema and infection to life-threatening obstruction and granulation tissue formation. Reported mortality rates in some studies have reached up to 6.2% [2]. Because there is no standardized algorithm for management, patient care must be individualized depending on the nature, size, and location of the foreign body, as well as the status of the airway [3].

Managing laryngeal foreign bodies presents several challenges, particularly in determining the appropriate surgical technique and anaesthetic method, ranging from intravenous or inhalational sedation to general anaesthesia, with or without tracheostomy. As a result, treatment strategies must be tailored to the specifics of each case. General anaesthesia should be used for the removal of tracheobronchial foreign bodies as it provides better control of the airway with high oxygen flow support and makes the patient inactive, thus reducing the risk of unnecessary injury to the oral cavity, larynx, and lower tracheobronchial tree. It also lowers metabolic demands during the procedure and allows for repeated and more thorough examinations, especially in the paediatric age group. If, however, upper airway obstruction is suspected, general anaesthesia should not be used because of the increased risk of total obstruction of the airway in these patients [7]. Maintenance of spontaneous ventilation during induction is recommended to prevent complete obstruction in partially compromised airways [10]. 

## Conclusions

Laryngeal foreign body impaction in adults is uncommon and may present with minimal symptoms such as isolated dysphonia. The nature of the foreign body in the larynx is variable and may be associated with the occupational environment and habits of the patient. This case highlights the importance of maintaining a high index of suspicion in atypical presentations and reinforces the role of prompt radiological evaluation, endoscopic removal to prevent potential airway complications and the value of a multidisciplinary approach, including anesthesiologists and otolaryngologists, in ensuring safe removal and optimal patient outcomes.
